# The effect of lifting load on the kinematic characteristics of lumbar spinous process in vivo

**DOI:** 10.1007/s00276-023-03135-6

**Published:** 2023-04-03

**Authors:** Huanxiong Chen, Zhenhao Zhong, Wangqiang Wen, Haoxiang Xu, Guojun Li, Tian Su, Zepei Zhang, Jun Miao

**Affiliations:** 1 Department of Spine Surgery, Hainan Province Clinical Medical Center, The First Affiliated Hospital of Hainan Medical University, Haikou, Hainan People’s Republic of China; 2 Department of Orthopaedics and Traumatology, SH Ho Scoliosis Research Laboratory, The Chinese University of Hong Kong, Shatin, NT, Hong Kong SAR, People’s Republic of China; 3 Joint Scoliosis Research Centre, Chinese University of Hong Kong and Nanjing University, The Chinese University of Hong Kong, Shatin, NT, Hong Kong SAR, People’s Republic of China; 4 The Second People’s Hospital of Hefei, Anhui, People’s Republic of China; 5 Department of Spine Surgery, Tianjin Hospital, Jiefangnanlu 406, Hexi District, Tianjin, 300210 People’s Republic of China

**Keywords:** Spinous process, In vivo, Loads, Kinematics, Range of motion

## Abstract

**Background:**

There are limited data on the in vivo natural kinematics of the lumbar spinous process. This paper intends to explore the effect of lifting load on the in vivo movement mode of the lumbar spinous process and its biomechanical changes.

**Methods:**

Ten asymptomatic subjects between the ages of 25 and 39 underwent CT scans of the lumbar spine in the supine position, and 3D models of L3-L5 were constructed. Using a Dual Fluoroscopy Imaging System (DFIS), instantaneous orthogonal fluoroscopic images of each subject's flexion–extension, left–right bending, and left–right rotational movements were taken under different loads (0 kg, 5 kg, 10 kg). The supine CT model was matched, using computer software, to the bony contours of the images from the two orthogonal views, so that the instantaneous 3D vertebral position at each location could be quantified. A Cartesian coordinate system was ultimately constructed at the tip of the spinous process to obtain the 6DOF kinematic data of the spinous process.

**Results:**

In different postural movements of the trunk, there was no significant difference in the rotation angle and translation range of the lumbar spinous process under different loads (P > 0.05). In flexion to extension motion, spinous processes mainly rotate < 4° along the medial and lateral axes and translate < 4 mm along the craniocaudal direction. In the left–right bending motion, spinous processes mainly rotate < 5° along the anterior and posterior axes, and the translation is mainly coupling < 2 mm. In the rotational motion, the spinous process is mainly coupled motion, the rotation range is less than 3°, and the translation range is less than 2 mm. The distance between spinous processes measured in the supine position was 6.66 ± 2.29 mm at L3/4 and 5.08 ± 1.57 mm at L4/5.

**Conclusion:**

The in vivo kinematics of the lumbar spinous process will not change significantly with increasing low load. In complex motion, the spinous process is dominated by coupling motion.

## Introduction

The kinematic characteristics of the lumbar spine have been the focus of research. Previous studies on the lumbar spine are mainly focused on the anterior structures of the spine, such as the vertebral body [17, 19], intervertebral disc [26], and facet joint [11, 23]. A few scholars have focused on the spinous process, which can increase the firmness and stability of the spine [3, 10]. Currently, research is directed toward the use of interspinous implants to treat the degenerative lumbar spine diseases [13, 28]. Therefore, the study of the in vivo movement mode of the spinous process can help improve the evaluation of traumatic injuries and degenerative changes in the posterior elements, as well as improve the surgical treatment of spinal diseases using posterior procedures.

According to previous studies, research on the lumbar spinous process mainly focuses on in vitro cadaveric research and in vivo X-ray and CT imaging research. Cadaveric research [6, 7] mainly uses anatomical experiments of human specimens to obtain the relevant anatomical parameters of spinous processes in different populations. Imaging studies, such as X-ray [7, 16] and CT [20], indirectly measure the interspinous process (ISP) distance between the lumbar levels to observe changes in the spinous process bone structure or surrounding soft-tissue ligaments and explore the best placement position of spinous process implants. However, there are few three-dimensional kinematic data about spinous processes in vivo.

The dual fluoroscopy imaging system (DFIS) is a new noninvasive in vivo imaging technology that can reproduce the instantaneous motion posture of human bones in vivo. At present, it can be widely used in research on the lumbar spine [14], knee joint [21], shoulder joint [12], and so on. This technology has the advantages of high accuracy, repeatability, and authenticity and few equipment limitations [22]. Li Guoan's team [22] played a series experiments which showed that the repeatability of the method in reproducing in vivo human spine 6DOF kinematics was less than 0.3 mm in translation and less than 0.7 degrees in orientation. Our team verified the accuracy and repeatability of this technique, with translation less than 0.43 mm and rotation less than 0.65°, which can be used to noninvasively measure spinal movement in vivo [2].

Li Guoan’s team [22] was the first to use the DFIS to study the kinematics of the cervical spine and lumbar spine in vivo. They are also the only team who has used this technology to study the three-dimensional kinematics of the spinous process in vivo under physiological load and provide accurate data of the lumbar ISP distance [25]. Not only that, they had described the characteristics of lumbar spinous process motion in degenerative disc disease and degenerative spondylolisthesis [27]. At present, no one has studied the in vivo biomechanical characteristics of spinous processes under weight-bearing conditions. It is noteworthy that, in Chowdhury’s research [5], it is found that the lifting load, especially when the lifting load is 13.5 kg, has a significant impact on the movement of lumbar facet joints, which is reflected in the increase of rotation and translation movement. Another research showed that heavy lifting (over 25 kg) and trunk flexion and rotation are considered to be moderate risk factors for low back pain [9].

Therefore, the purpose of this research is to study the effect of lifting load (0 kg, 5 kg, 10 kg) on the kinematics characteristics of the lower lumbar spinous process by DFIS. To obtain the data of six degrees of freedom of the lumbar spinous process in asymptomatic participants under weight-bearing conditions. We hypothesize that the in vivo kinematics of the lumbar spinous process are affected by weight-bearing conditions and vertebral level.

## Methods

### Participants

Ten asymptomatic subjects (5 males and 5 females, age 32 ± 4 y, height 1.67 ± 0.09 m, weight 62.75 ± 10.30 kg, BMI 22.32 ± 2.16 kg/m^2^) were recruited (Table 1). Inclusion criteria: (1) normal spine development and unrestricted lumbar movement; (2) no previous symptoms of low back pain, lumbar trauma, or surgical history; (3) normal BMI, 18.5 ≤ BMI ≤ 25; (4) Pfirrmann classification of intervertebral disc degeneration ≤ grade II (MRI); (5) bone mineral density is in the normal range. The exclusion criteria were as follows: (1) spinal deformity and anatomical abnormalities, such as spina bifida and isthmus; (2) previous symptoms of low back pain, history of lumbar trauma, and lumbar surgery; (3) BMI > 25 or BMI < 18.5; (4) Pfirrmann grade > grade II; (5) severe osteoporosis and other spinal diseases affecting the experiment; and (6) pregnancy and childbirth.

**Table 1:** General information on recruitment of volunteers

Volunteers	Gender	Age	Height (m)	Weight (kg)	BMI (kg/m^2^)	Pfirrmann
1	Male	25	1.72	58	19.6	I
2	Male	25	1.69	64	22.4	I
3	Male	35	1.72	74	25	I
4	Male	34	1.75	74.5	24.3	I
5	Male	31	1.82	79	23.8	I
6	Female	39	1.6	57	22.3	I
7	Female	32	1.58	59	23.6	I
8	Female	33	1.56	57	23.4	I
9	Female	32	1.56	45	18.5	I
10	Female	34	1.72	60	20.3	I
Total	Mean	32.00	1.67	62.75	22.32	
	SD	4.29	0.09	10.30	2.16	

This study was approved by the medical ethics committee of Tianjin Hospital, and each subject signed an informed consent form before the experiment.

### 3D modelling technology

**Fig. 1:**
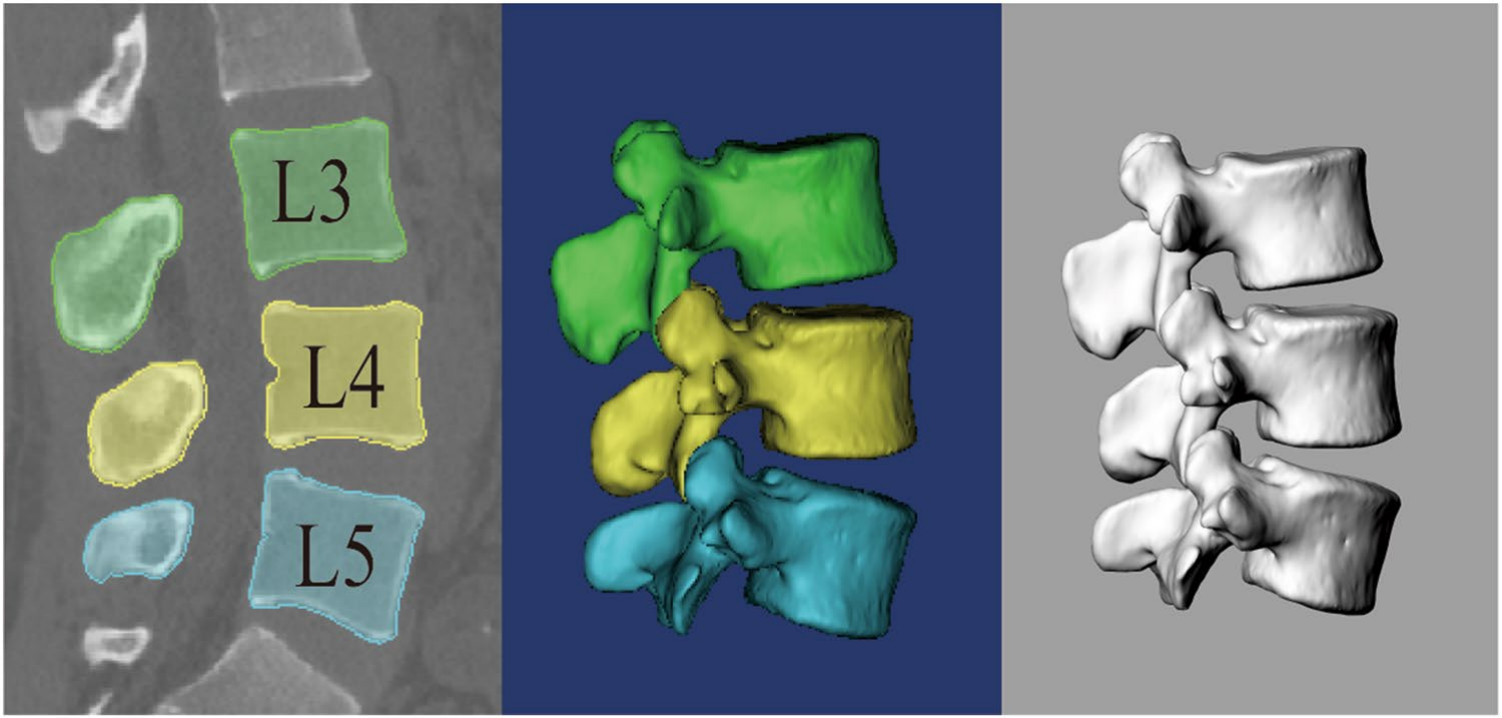
Establishment of the L3–L5 vertebral body in rhino

Before the experiment, the subjects lay flat on the bed and were in a state of full rest and calm to ensure that the lumbar relaxation was closer to the actual physiological state. Then, high-resolution computed tomography (CT) (sensing 16 Siemens, Germany) was performed in the supine position, and a medical digital imaging and communication format (DICOM) with a thickness of 0.625 mm and a resolution of 512 * 512 pixels was obtained. The DICOM images were imported into the solid modelling software (mimics version 19.0), and the 3D anatomical vertebral body model of L3–L5 was constructed using the established and validated protocol [2]. After outputting the three-dimensional model into binary stereo lithography (STL) format, it is imported into the solid modelling software rhino (Rhinoceros® Robert McNeel & Associates, Seattle, Washington) for further processing and data acquisition (Fig. 1).

### Dual fluoroscopy imaging system (DFIS)

**Fig. 2:**
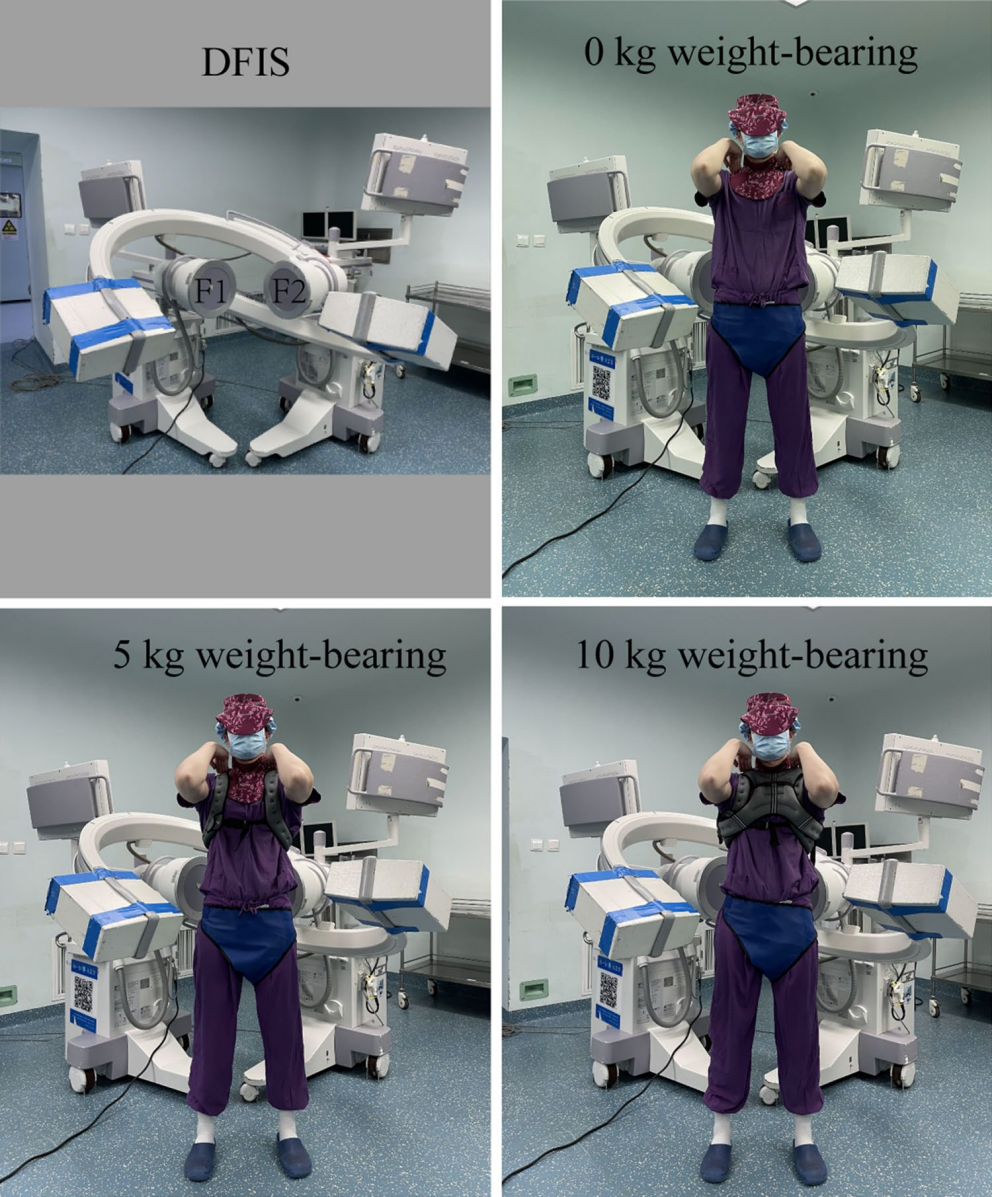
**a** Dual fluoroscopy imaging system. **b**, **c,** and **d** are 0 kg, 5 kg, and 10 kg loads, respectively. F1 and F2 are fluorescent mirrors perpendicular to each other

**Fig. 3:**
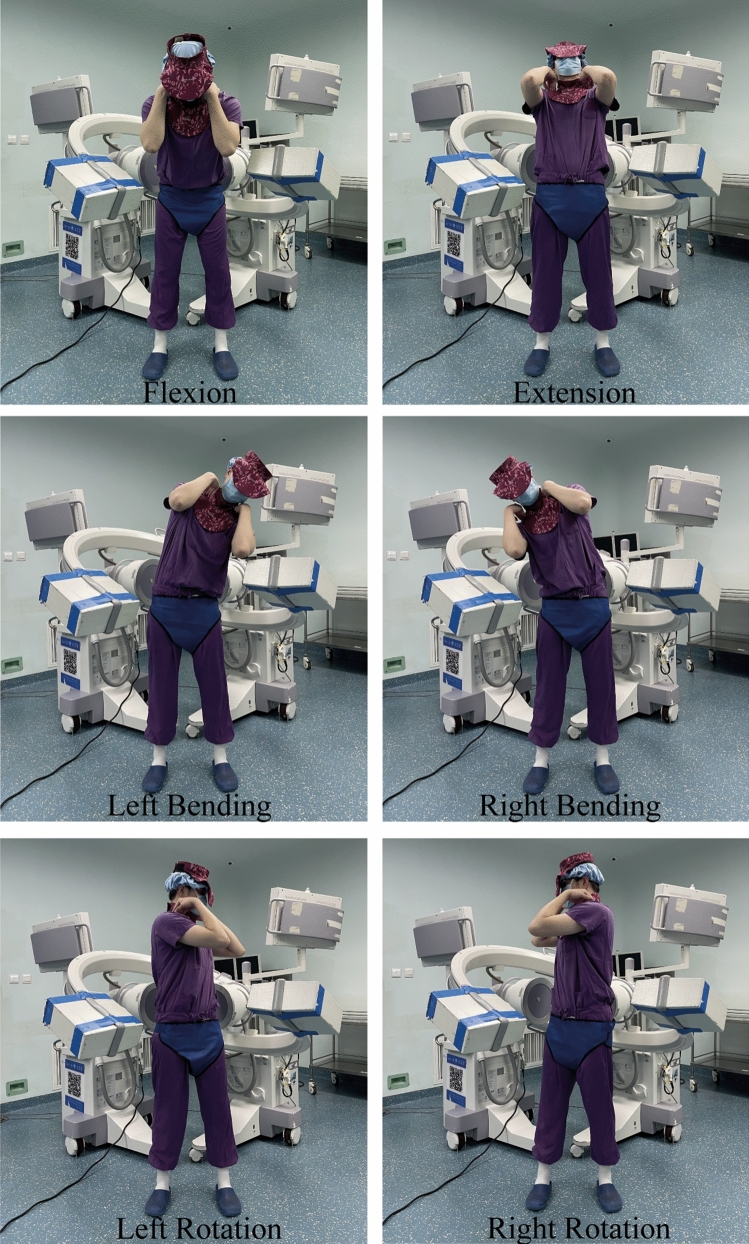
Mode of motion

The perspective imaging system is composed of two "C" arm X-ray machines of the same model and perpendicular to each other (Fig. 2a, fluorescent mirrors of F1 and F2 perpendicular to each other). Before the experiment, the previously customized DFIS calibration system instrument was used to debug the accuracy of the equipment to reduce the error [2]. According to the established loads of 0 kg, 5 kg (carrying 5 kg sandbags) and 10 kg (carrying 5 kg sandbags at the front and rear) (Fig. 2b, c and d), flexion to extension, left–right bending, and left–right rotation were performed (Fig. 3). Because the volume, height, and diameter of the lumbar intervertebral disc are significantly changed after a day's activity, volunteers need to rest fully before the experiment, and the interval between different weight-bearing periods was 10 min to simulate the state after lumbar activity. For each subject, the X-ray image was required to stay for approximately 2 s. The whole experiment was guided by two professional spine surgeons and assisted in restraining the hip and knee joints to reduce the errors caused by other joint activities. At the same time, observe and ensure that the L3–L5 segment of the subject is always within the orthogonal projection range of the two fluoroscopes during movement. Before shooting, we wore customized lead clothes to protect the subjects’ thyroid and gonads.

### 3D reconstruction of lumbar internal motion

**Fig. 4:**
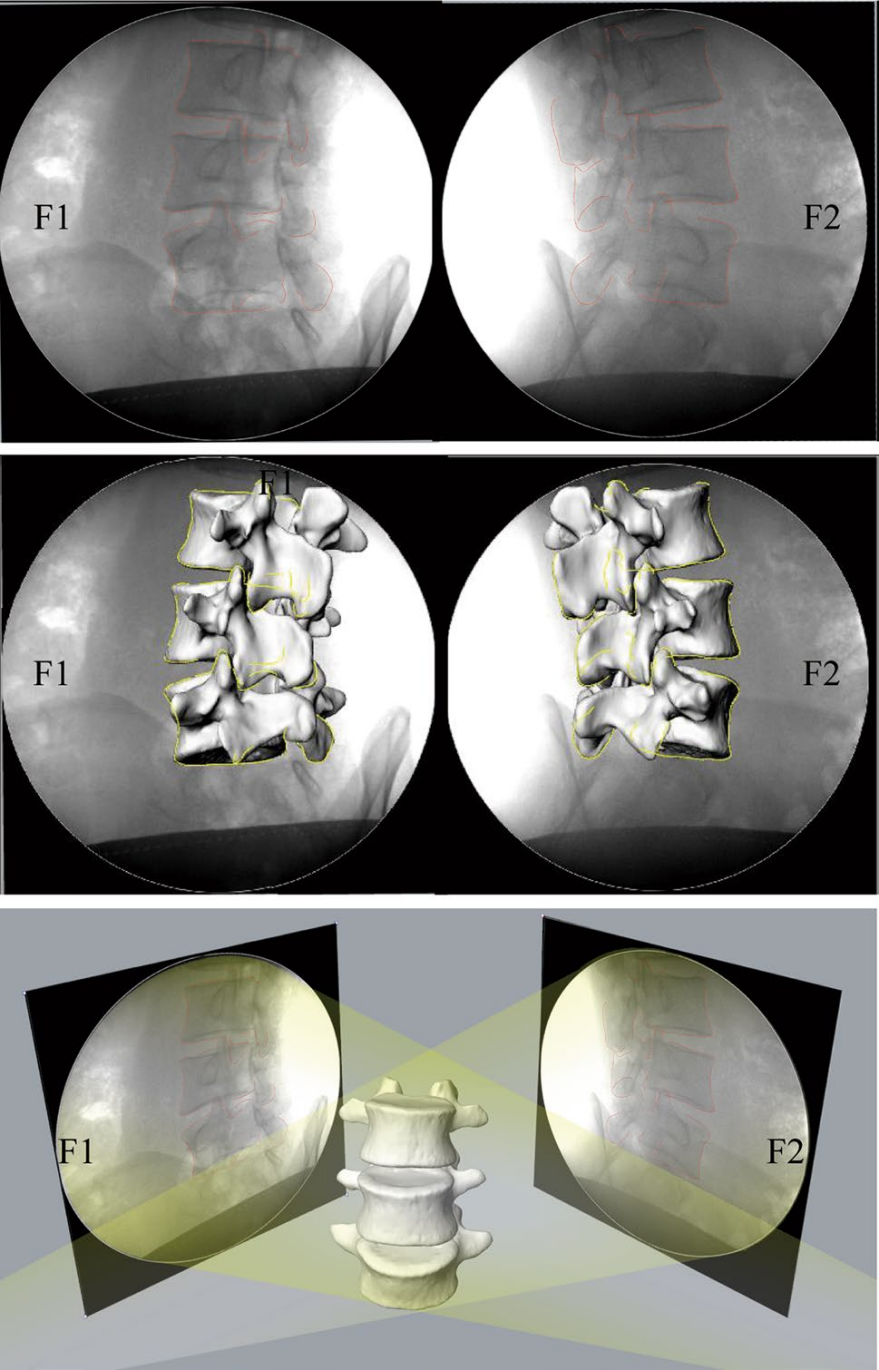
**a** Outline of the vertebral structure. **b**, **c** Make the vertebral body fully match the perspective image. F1 and F2 are fluorescent mirrors perpendicular to each other

Using 3D modelling technology based on a double fluoroscopy imaging system (DFIS) combined with CT, the instantaneous in vivo motion of the vertebral body can be reproduced in Rhinoceros modelling software [2, 22]. First, the previously obtained orthogonal X-ray images are introduced into the Rhinoceros software, and then, the lumbar anatomical structures, such as the vertebral body, facet joint, and spinous process, are outlined (Fig. 4a). Finally, after translating and rotating the spatial position of the lumbar model, the three-dimensional lumbar model matched the two orthogonal X-ray fluoroscopy images (Fig. 4b), simultaneously, to realize the matching of two-dimensional and three-dimensional images (Fig. 4c).

### Spinous process kinematics measurement

**Fig. 5:**
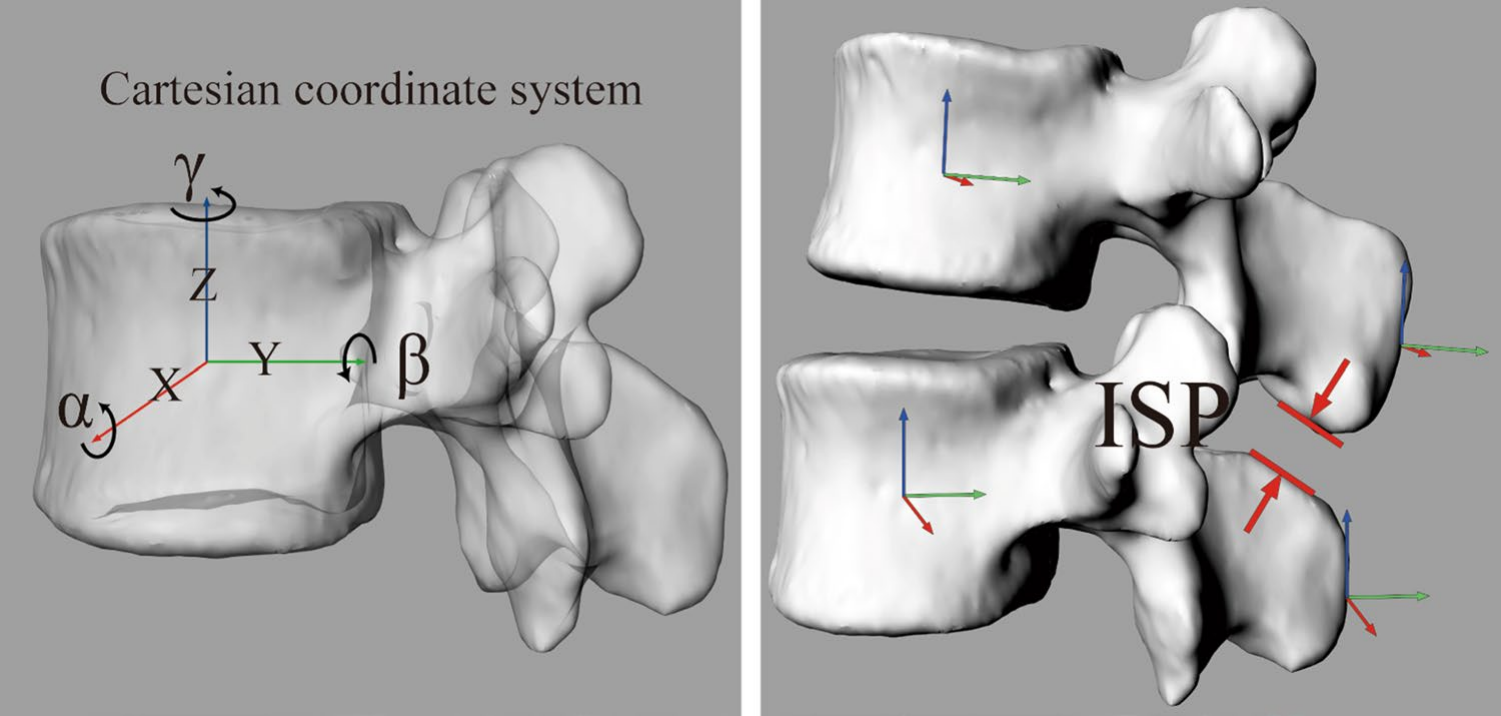
**a** Establishes a standard Cartesian coordinate system. *X*(red): The line perpendicular to the sagittal plane of the cone, and points to the left. *α*: The rotation angles along the *X* axis. *Y*(green): The line parallel to the sagittal plane of the cone and points to the back. *β*: The rotation angles along the *Y* axis. *Z* (blue): The line perpendicular to the coronal plane of the cone and points to the cephalic side. *γ*: The rotation angles along *Z* axis. **b** Move the standard Cartesian coordinate system to the tip of the spinous process. ISP, interspinous process

In the supine position, establish a standard Cartesian coordinate system (Fig. 5a) at the centre of the vertebral body of the standard L3/L5 model, define that the *X* axis (red) is perpendicular to the sagittal plane of the cone and points to the left, define that the *Y* axis (green) is parallel to the sagittal plane of the cone and points to the back, and the *Z* axis (blue) is perpendicular to the coronal plane of the cone and points to the cephalic side. The *X* (red), *Y* (green), and *Z* (blue) axes in the space coordinate represent the direction vector in the space. *α*, *β*, and *γ* are the rotation angles along the *X* axis, *Y* axis, and *Z* axis, respectively. Then, it is copied and moved to the tip of the spinous process; that is, the right-hand Cartesian coordinate system of the spinous process is created (Fig. 5b), and the 6DOF of the spinous process in space can be obtained. By calculating the difference between the two coordinates of adjacent spinous processes, the data of relative motion in the body are obtained. At the same time, we also measured the shortest vertical distance between spinous processes while maintaining the supine position in the rhino software, which was consistent with the anatomical characteristics of spinous processes (Fig. 5b). This method was accuracy [25, 27].

### Statistical analysis

Two-way repeated-measures ANOVA was used to compare the range of motion of the lumbar spinous process at the L3/4 and L4/51 vertebral levels under different weight-bearing conditions. Kinematics was the dependent variable, and weight-bearing and vertebral body level were the independent variables. The statistical significance level was set as *P* < 0.05. When statistically significant differences were detected, the Newman–Keuls post-test was performed. one-way analysis of variance was used to statistically analyses the rotation range and translation range of the spinous process in different directions, and LSD test was used for pairwise comparison; test level *α* = 0.05. All analyses were performed using SPSS software (SPSS for Windows, version 19.0; IBM, Armonk, NY, USA), with continuous variables expressed as X ± SD.

## Result

By applying DFIS combined with the method of CT, we measured the motion of the spinous process in vivo. This process is performed using existing protocols and technologies established in our laboratory [2], which can reproduce the 6DOF kinematics of human spines in vivo. The translation is less than 0.43 mm, and the rotation is less than 0.65°.

**Fig. 6:**
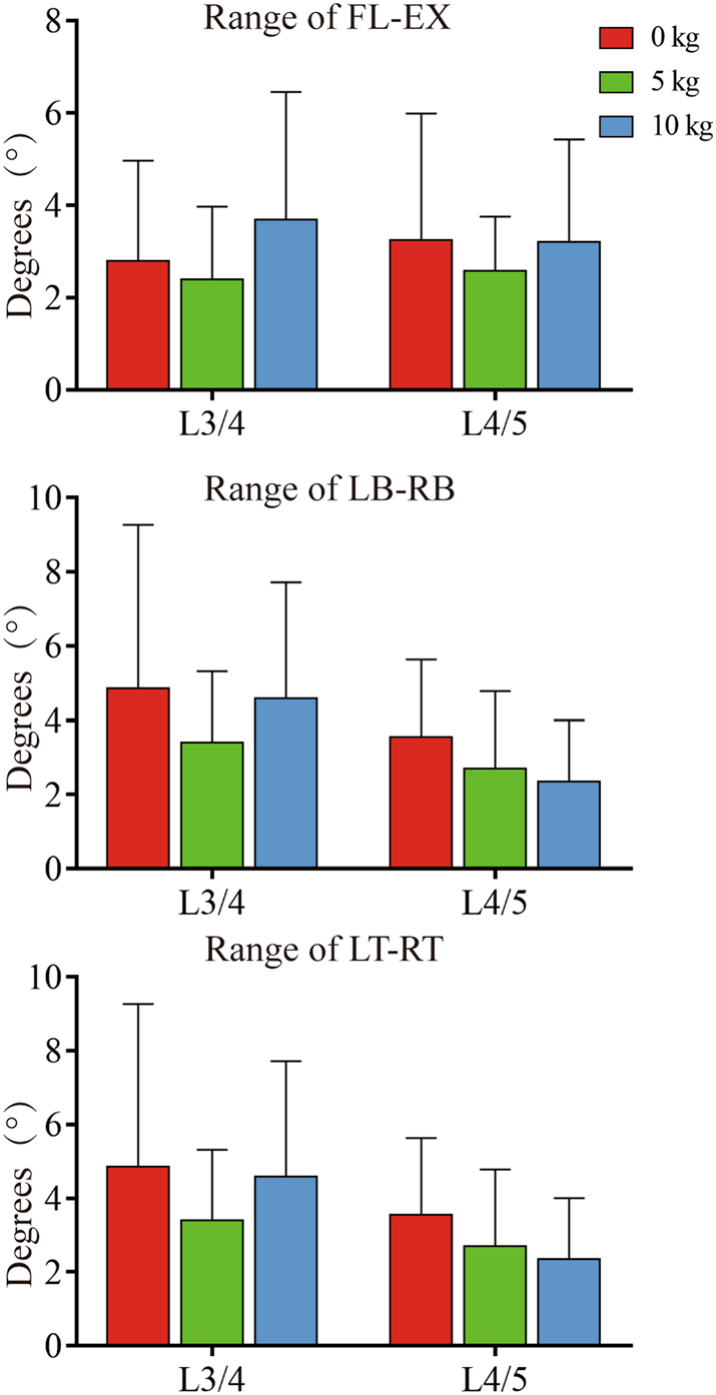
Rotation range of the primary rotation axis. FL–EX, flexion–extension. LB–RB, left–right bending. LT–RT, left–right rotation

In the flexion and extension of the trunk, the spinous process of the lumbar spine mainly rotates around the medial–lateral axis, and the range is less than < 4° (Table 2). The average rotation ranges of the horizontal main rotation axes of the L3/4 and L4/5 segments under different loads (0 kg, 5 kg and 10 kg) are 2.79 ± 2.18° to 3.68 ± 2.78° and 2.58 ± 1.81° to 3.25 ± 2.74°, respectively. The weight-bearing condition had no significant effect on the rotation range of the spinous process (*P* > 0.05) (Fig. 6). The data showed that there was no significant difference in the rotation range of the main rotation axis in different horizontal segments. The translation direction was mainly craniocaudal (*P* < 0.05), and the size was < 4 mm (Table 2), so weight-bearing had no significant effect on it.

**Table 2:** The internal rotation range of the spinous process under different loads (mean ± SD)

Position	Level	Loads	Rotation (°)			Translation (mm)		
			*α*	*β*	*γ*	*X*	*Y*	*Z*
FL-EX	L3/4	0 kg	2.79 ± 2.18	1.85 ± 1.71	1.32 ± 0.94	1.18 ± 1.12	0.51 ± 0.45	2.68 ± 2.65
		5 kg	2.39 ± 1.58	1.41 ± 1.24	1.12 ± 1.03	1.23 ± 0.85	0.47 ± 0.27	2.35 ± 1.81
		10 kg	3.68 ± 2.78	1.64 ± 1.68	1.5 ± 1.61	1.12 ± 1.02	0.53 ± 0.35	3.56 ± 2.66
	L4/5	0 kg	3.25 ± 2.74	1.22 ± 0.64	2.62 ± 2.49	1.48 ± 1.58	1.03 ± 0.79	3.08 ± 2.51
		5 kg	2.58 ± 1.81	1.16 ± 0.69	1.15 ± 0.44	0.97 ± 0.37	0.71 ± 0.63	2.6 ± 1.79
		10 kg	3.21 ± 2.22	1.25 ± 1.08	2.04 ± 1.86	1.67 ± 1.2	0.65 ± 0.22	3.04 ± 2.06
LB-RB	L3/4	0 kg	1.09 ± 0.96	4.85 ± 4.42	1.16 ± 1.15	1.15 ± 0.81	0.45 ± 0.23	1.26 ± 1.07
		5 kg	1.85 ± 0.82	3.41 ± 1.91	1.88 ± 0.98	1.33 ± 0.63	0.47 ± 0.42	1.85 ± 1.03
		10 kg	1.39 ± 0.76	4.59 ± 3.13	1.74 ± 0.63	1.2 ± 0.82	0.83 ± 0.41	1.3 ± 0.67
	L4/5	0 kg	1.25 ± 0.81	3.56 ± 2.08	1.22 ± 0.99	1.14 ± 0.75	0.7 ± 0.48	1.08 ± 0.72
		5 kg	1.46 ± 1.3	2.7 ± 2.09	1.54 ± 1.19	1.06 ± 0.86	0.65 ± 0.78	1.74 ± 1.92
		10 kg	1.16 ± 1.31	2.35 ± 1.85	1.33 ± 0.97	1.08 ± 1.17	0.58 ± 0.42	1.19 ± 1.23
LT-RT	L3/4	0 kg	0.93 ± 0.48	2.58 ± 1.65	1.33 ± 1.16	0.83 ± 0.93	0.43 ± 0.3	0.93 ± 0.75
		5 kg	1.57 ± 1.14	1.85 ± 1.37	1.74 ± 2.19	0.69 ± 0.68	0.49 ± 0.4	1.88 ± 1.24
		10 kg	1.18 ± 0.92	1.73 ± 1.3	1.29 ± 1.28	1.18 ± 0.92	0.62 ± 0.44	1.73 ± 1.11
	L4/5	0 kg	1.61 ± 1.16	2.52 ± 2.11	1.01 ± 0.75	0.55 ± 0.48	0.39 ± 0.27	1.65 ± 1.06
		5 kg	1.34 ± 0.94	1.59 ± 1.38	2.04 ± 2.31	1.08 ± 0.93	0.56 ± 0.28	1.65 ± 1
		10 kg	0.89 ± 0.69	2.64 ± 1.88	1.89 ± 2.13	1.13 ± 1.9	0.95 ± 0.86	0.92 ± 0.44

In the lateral bending movement, the spinous process of the lumbar spine mainly rotates around the anterior and posterior axes, and the range is less than 5° (Table 2). The average rotation ranges of the L3/4 and L4/5 horizontal main rotation axes under different loads (0 kg, 5 kg and 10 kg) are 3.41 ± 1.91° to 4.85 ± 4.42° and 2.35 ± 1.85° to 3.56 ± 2.08°, respectively. The data showed that the rotation range of the spinous process decreased with increasing load, but there was no significant difference (*P* > 0.05) (Fig. 6). Translation has no main direction and is dominated by coupling motion (*P* > 0.05). The translation range in all directions is less than 2 mm (Table 2), and lifting the load has no significant effect on it.

In rotational motion, the primary rotational axis of the lumbar spinous process is the craniocaudal axis, but the data show that spinous process in vivo motion is a complex coupling motion (Table 2). The average rotation ranges of the horizontal main rotation axes of the L3/4 and L4/5 segments under different loads (0 kg, 5 kg and 10 kg) are 1.29 ± 1.28° to 1.74 ± 2.19° and 1.01 ± 0.75° to 2.04 ± 2.31°, respectively. We did not find that weight-bearing led to significant changes in the rotational kinematics of lumbar facets (*P* > 0.05) (Fig. 6). The translation had no main direction (*P* > 0.05), and the translation range in each direction was less than 2 mm (Table 2). Lifting the load has no significant effect on it.

In addition, we also directly measured the shortest distance between spinous processes in the supine position in the modelling software. At L3/4, it was 6.66 ± 2.29 mm, and at L4/5, it was 5.08 ± 1.57 mm. There was no difference between segments (*P* > 0.05) (Table 3).

**Table 3:** Shortest distance between processes in supine position (mean ± SD)

Positions	Level	
	L34	L45
Supine	6.66 ± 2.29 mm	5.08 ± 1.57 mm

## Discussion

In this experiment, we measured the kinematic changes the in spinous processes of the lumbar segments under different functional loads (0 kg, 5 kg, 10 kg). We found that the rotation angle and translational motion of the spinous process did not change significantly between different load conditions, indicating that low load conditions are not the main factors affecting rotation and translation. Complex motions, such as side-bending and rotation, are mainly reflected in coupling motion. In our published study [8], it was also found that the rotational motion of lumbar joints in sitting posture is dominated by coupled motion, which can provide a new perspective for lumbar kinematics.

In our results, the ISP distance in the supine position was 6.66 ± 2.29 mm at L3/4 and 5.08 ± 1.57 mm at L4/5. In Li Guoan's study [25], the results were 7.5 ± 3.0 mm and 5.6 ± 3.0 mm, respectively. In Lin’s study [15], it was found that the distance between spinous processes in the middle of the spinous process was the shortest, 7.79 ± 3.80 mm at L3/4, and 6.45 ± 3.38 mm at L4/5. Our results are almost consistent with their results; there is no difference between segments, which can also verify the accuracy of our experiment.

In our published studies [23, 26], the coupling translation part of intervertebral disc compression deformation and facet scoliosis were affected by an additional 10 kg load, while research on spinous process structure had no significant effect on load movement. This finding suggests that in spinal movement, the vertebral body and intervertebral joints conduct and limit spinal movement and bear most of the load. The spinous process can further strengthen and stabilize the spine under the action of surrounding tissues and ligaments and jointly maintain the stable movement of the body. If the load is large or abnormal, the interspinous processes collide and squeeze the interspinous ligament. When the interspinous ligament has been repeatedly squeezed for a long period time, it breaks and degenerates, which causes the extrusion surface of the spinous process to harden, proliferate, and become hypertrophied, thus resulting in Baastrup's disease [1, 6].

According to previous studies, with the increasing incidence of lumbar degenerative disease (LDD) each year, people began to compare the transformation of the anterior part of the spine to the posterior column. Cai [3] and Ihm [10] studied the spinous process morphology of Chinese and Korean populations, respectively, and found that there were differences in the spinous process morphology between different ages and sexes. Neumann [16] studied the X-ray photos of 200 normal thoracolumbar vertebrae, which showed that the distance between adjacent spinous processes is more than 7 mm, so there may be damage to the posterior column structure of the spine, which greatly increases the probability of lumbar instability. Li Guoan's team [27] used DFIS to observe ISP changes of the spinous processes in patients with DDD and DLS, and found that the spinous processes in patients with DDD were hyperactive and that LSP activities in patients with DLS were insufficient. These data may help to improve complications such as ISP hand fracture and device dislocation. In Lin's study [15], they observed a decreasing tendency in interspinous distance from L2–3 to L5–S1 in the supine, standing, flexion, and extension postures by measuring the interspinous distance in different postures. Our study provided the kinematic data of weight-bearing movement in the spinous process and found that mild weight-bearing did not cause pathological activity of the spinous process. This is a further addition to spinous process knowledge, which is very important for understanding spinal pathology, the before-and-after effects of the spinous process operation and postoperative rehabilitation training.

Currently, research is directed toward the use of interspinous implants to treat spinal canal stenosis or facet arthritis and other related diseases. Interspinous implants are used to reduce the burden on facet joints and restore the height of the intervertebral foramen to maintain the motion stability of the lumbar posterior column [24]. Different types of interspinous implants have different designs and are made of different materials. They have different biomechanical effects on internode kinematics, such as range of motion and rotation centre [18, 24]. Therefore, many influencing factors should be considered in the design of interspinous implants, such as patient age, BMI, bone mineral density, sex, spinous structure, lumbar disease, and weight-bearing [4].

## Limitations

Our study also has some limitations. First, the experimental process of this study involved matching of the vertebral bodies, which is a difficult and time-consuming process. In the future, we should further study the automatic matching method. Second, due to the limitation of fluoroscopy, our motor segments are only limited to L3 / 5. We will try to study the whole lumbar segment in the future. Third, the relationship between vertebral structures, such as the intervertebral disc, facet joint, and spinous process, should be further discussed. In the future, the common change trend between vertebral structures should be further studied by the finite element method. Despite the above limitations, our study still provides accurate data on the in vivo movement of the lumbar spinous process in different postures under weight-bearing conditions.

## Conclusion

In conclusion, this study provides quantitative data on spinous process movement under various weight-bearing postures. Theoretically, this information can guide the design of related interspinous implants, which is very important to determine the surgical method needed to treat spinous process disease, improve the surgical effect, and accelerate rehabilitation.

## Data Availability

The data sets used and/or analyzed during the current study are available from the corresponding author on reasonable request.

## Abbreviations

DFIS: Dual fluoroscopy imaging system
ISP: Interspinous process
6DOF: 6 Degrees of freedom
ROM: Ranges of motion
DICOM: Digital imaging and communications in medicine
